# Eight Letters with Replies

**Published:** 1877-07

**Authors:** 


### Our Correspondence.

DOWNSVILLE, N. Y., March 28, '77.

DR. UP DE GRAFF:

I have been an attentive reader of your valuable BISTOURY for some years, and seeing you receive correspondence from many persons afflicted with disease, I concluded you would notice my short letter. I was burned some six or seven years ago by an explosion of powder, which left some red scars about my face, which I would give most any price to have removed. If you could give me any advice or send me any receipt for their removal, I would pay you your price willingly. C.

If the scars are broad and red, they can be removed surgically, and new skin transplanted. If not very large, had better let them alone.

---

NASHVILLE, TENN., April 18, 1877.

DR. UP DE GRAFF:

My father has what I suppose to be *cataract*. He is seventy-one years old, and his sight has been gradually failing him for three years. He is now entirely blind in his right eye and nearly so in his left. Can he have his right eye operated upon now, or must he wait until he is blind in his left one, and have both done at once? Can we come at *any* time to have the operation performed, or should we select cold weather as the best time? J. R. B.

He can have the blind eye operated upon at any time—need not wait for the second one to become blind. The operation can also be done at any season of the year that suits your convenience.

---

GREEN, N. Y., 4, 29, 1877.

DR. UP DE GRAFF:

*Dear Sir:*—I admire a forehead with an open space between the eyebrows, and would know is there a harmless recipe for removing the hair and trimming the eyebrows. Will pulling out the hair for a number of times have the desired effect? C. T. B.

Pulling out the hairs will not prevent their regrowing. See back numbers of the BISTOURY for a depilatory that will answer your purpose.

---

JACKSON, Mich., May 7th, 1877.

DR. UP DE GRAFF:

Our little boy, aged four, has a rupture. The tumor seems to grow larger. Is there anything we can do to stop it? P. R.

Construct a snug-fitting bandage, to go about his waist, with gores cut to fit the top of the hips. Take an old elastic suspender, cut two pieces from it, each two or three inches long. Sew them to each end of a padded band, long enough to reach between the legs and button snugly upon the waist-band, front and rear. To the band passing between the limbs, fasten a smooth rubber button, as large as half of a black walnut. Let this rest immediately over the opening in the abdomen through which the intestine is found to protrude. This will hold the hernia in place, and if worn faithfully for a year, is likely to cure the boy. Better have your family physician apply the bandage and advise you in its construction.

---

FRANKFORT, Ky., May 17th, 1877.

EDITOR BISTOURY:

What can I do for my little boy, six years of age, who is suffering from paralysis of both legs? He can stand alone but cannot walk. MRS. L.

Cannot tell without knowing more about the case. See last number of the BISTOURY for full information upon this subject.

---

OWATONNA, Minn., June 1st, 1877.

DR. THAD S. UP DE GRAFF:

Have read the BISTOURY for many years, have been interested and entertained by it, but now am forced to seek its advice. Have been using my eyes considerably lately in reading, particularly in the evening, to entertain an invalid mother. I am now troubled with sensitiveness to light, with bright and dark specks floating before my eyes. Now and then pain is felt in the eyes and over the brow. They are growing worse, so that it is now difficult for me to read at all. What can I do for them? MRS. J. D.

Stop reading, writing, sewing or other work in which the eyes are employed. Give your eyes absolute and complete rest. Bathe the closed eyelids in cold water several times daily. Keep cool and quiet. If the unpleasant symptoms do not speedily disappear, consult an oculist. You are suffering from congestion of the retina, brought on by over-use of the eyes.

---

TUSCALOOSA, Ala., June 1st, 1877.

DR. UP DE GRAFF:

Where can I procure one of Dr. Knapp's ophthalmoscopes? J. R. P., M. D.

Of Geo. Tiemann & Co., 67 Chatham St., N. Y.

---

SUNBURY, Pa., June 2d, 1877.

THAD S. UP DE GRAFF, M. D.:

Can you recommend any tolerably sure cure for diphtheria? Several deaths have occurred here from this dreadful disease, and we are becoming considerably alarmed over it. S.

Read Medical Items and News, present number, for the most approved means of treatment.

